# A Faith to Move Mountains? Childhood Abuse, Religious Change, and Mental Health at Midlife

**DOI:** 10.1093/geroni/igaa057.1493

**Published:** 2020-12-16

**Authors:** Laura Upenieks

**Affiliations:** University of Texas at San Antonio, San Antonio, Texas, United States

## Abstract

Of all the various forms of adversity experienced during childhood, childhood maltreatment (emotional and physical abuse) is shown to have the largest impacts on mental health and well-being. Yet we still have a limited understanding of why some victims of early maltreatment suffer immense mental health consequences later on in the life course, while others are able to cushion the blow of these early insults. Using two waves of data from the National Survey of Midlife Development in the United States (MIDUS), this study considers change in religiosity as a buffer across three dimensions for victims of childhood abuse: religious importance, attendance, and the specific act of seeking comfort through religion. Results suggest that increases in religious comfort during adulthood are positively associated with adult mental health for victims of abuse, while decreases in religious comfort over time were associated with worse mental health. Changes in religious attendance and religious importance were not significant associated with mental health for victims of abuse. Taken together, my results show that the stress-moderating effects of religion for victims of childhood maltreatment are contingent on the stability or increases or decreases in religiosity over the life course, which has been overlooked in previous work.

